# Lifespan and Glucose Metabolism in Insulin Receptor Mutant Mice

**DOI:** 10.4061/2011/315640

**Published:** 2011-08-18

**Authors:** Takahiko Shimizu, Tomonori Baba, Midori Ogawara, Takuji Shirasawa

**Affiliations:** ^1^Molecular Gerontology, Tokyo Metropolitan Institute of Gerontology, 35-2 Sakae-cho, Itabashi-ku, Tokyo 173-0015, Japan; ^2^Applied Biological Chemistry, United Graduate School of Agricultural Science, Tokyo University of Agriculture and Technology, Fuchu-shi, Tokyo 183-8509, Japan; ^3^Department of Aging Control Medicine, Juntendo University Graduate School of Medicine, Bunkyo-ku, Tokyo 113-0033, Japan

## Abstract

Insulin/insulin-like growth factor type 1 signaling regulates lifespan and resistance to oxidative stress in worms, flies, and mammals. In a previous study, we revealed that insulin receptor (IR) mutant mice, which carry a homologous mutation found in the long-lived *daf*-2 mutant of *Caenorhabditis elegans*, showed enhanced resistance to oxidative stress cooperatively modulated by sex hormones and dietary signals (Baba et al., (2005)). We herein investigated the lifespan of IR mutant mice to evaluate the biological significance of insulin signaling in mice. Under normoxia, mutant male mice had a lifespan comparable to that of wild-type male mice. IR mutant female mice also showed a lifespan similar to that of wild-type female mice, in spite of the fact that the IR mutant female mice acquired more resistance to oxidative stress than IR mutant male mice. On the other hand, IR mutant male and female mice both showed insulin resistance with hyperinsulinemia, but they did not develop hyperglycemia throughout their entire lifespan. These data indicate that the IR mutation does not impact the lifespan in mice, thus suggesting that insulin signaling might have a limited effect on the lifespan of mice.

## 1. Introduction

Accumulating evidence indicates that insulin/insulin-like growth factor type 1 (IGF-1) signaling regulates lifespan in worms, flies, and mammals [1, 2]. In *Caenorhabditis elegans*, a mutation of the *daf-2* gene that encodes an insulin/IGF-1 receptor ortholog significantly extended the lifespan and enhanced the resistance of the worms to oxidative stress [3, 4]. The lifespan extension caused by *daf-2* mutations required the activity of *daf-16* [3], which encodes a FOXO family transcription factor [5, 6]. Insulin/IGF-1 receptor mutations can also increase the lifespan of *Drosophila* [7]. In addition, mutations in *chico,* a downstream insulin receptor (IR) substrate-like signaling protein, increased the lifespan of the flies [8]. In mice, long-lived hereditary dwarf mice have been described [9]. Low levels of circulating growth hormone (GH) and IGF-1 in the Ames and Snell dwarf mice, which have pituitary defects, were associated with an extension of their lifespan [9]. Mutations in upstream genes that regulate insulin and IGF-1 also extended lifespan. For example, *Little* mice harbor a mutation in the GH-releasing hormone receptor and display reduced GH, as well as prolactin secretion [10]. *Little* mice also show reduced IGF-1 in blood, and have an increased mean and maximal lifespan [9]. Furthermore, GH receptor (GHR) mutant mice showed reduced circulating IGF-1 levels and an increased lifespan [11]. 

Although insulin/IGF-1 signaling controls the lifespan in mice, global deletion of insulin or IGF-1 causes early lethality associated with severe growth retardation. Mice lacking two nonallelic insulin genes, *Ins1* and *Ins2*, died in the early postnatal stage [12], while more than 95% of IGF1-null mice died perinatally [13]. Unlike worms and flies, which have a single insulin/IGF-1-like receptor, mice have separate receptors for insulin and IGF-1. Mice with a global deletion of either the IR or the IGF-1 receptor (IGF-1R) gene showed early postnatal lethality [14–16]. However, female mice with a heterozygous deletion of the IGF-1R displayed a 26% increase in mean lifespan [17]. IGF-1R heterozygous mice also showed mild growth retardation, but normal IGF-1 levels, as well as enhanced resistance to oxidative stress. In addition, mice that lack the IR gene in adipose tissue (FIRKO) live significantly longer than wild-type mice [18]. 

In a previous study, we generated a homologous murine model by replacing the Pro^1195^ of IR with Leu^1195^ using a targeted knock-in strategy to investigate the biological significance of longevity mutations found in the *daf-2* mutant of *C. elegans *[19]. The homozygous mice died during the neonatal stage from diabetic ketoacidosis [19, 20], which was consistent with the phenotypes of IR-deficient mice [14, 16]. On the other hand, heterozygous mice showed suppressed kinase activity of the IR, but they grew normally without spontaneously developing hyperglycemia during adulthood. Furthermore, we demonstrated that IR mutant (*Ir* ^*P*1195*L*/wt^) mice acquired an enhanced resistance to oxidative stress, such as exposure to 80% oxygen or paraquat. We also revealed that gender differences and dietary restriction are also associated with the defective insulin signaling [19]. 

In the present study, we investigated the lifespan of *Ir* ^P1195L/wt^ male and female mice under normoxia to clarify the insulin signaling resulting from a homologous mutation of *daf-2* as a determinant of mammalian lifespan. Furthermore, we investigated the pathological consequences of the IR mutation related to systemic insulin resistance, because insulin signaling is generally regulated to the glucose metabolism in mammals. We herein reveal that the *Ir* ^P1195L/wt^ mice showed a normal lifespan and did not develop hyperglycemia, as is seen in diabetes mellitus (DM) patients. 

## 2. Materials and Methods

### 2.1. Animals

*Ir* ^P1195L/wt^ mice [19] were backcrossed to C57BL6/NCrSlc mice (Japan SLC, Hamamatsu, Japan) for five or six generations. Mice were housed in specific pathogen-free facilities on a 12 h light/dark cycle (0800 on, 2000 off) and were fed an autoclaved standard chow (CRF-1; Oriental Yeast, Tokyo) and water *ad libitum*. The chow consisted of 54.5% (wt/wt) carbohydrate, 5.7% (wt/wt) fat, 22.4% (wt/wt) protein, and 3.1% (wt/wt) dietary fiber. Food intake was measured on a monthly basis until death. For measurement of the reproduction rate, we mated females with fertile males for four weeks at sexual maturity, and recorded the resulting pregnancies and offsprings. For measurement of rectal temperature, we used a digital thermometer with a rectal probe (BDT-100, Bio Research Center, Nagoya, Japan). All protocols for animal use and experiments were reviewed and approved by the Animal Care Committee of the Tokyo Metropolitan Institute of Gerontology. 

### 2.2. Lifespan Determination

By mating *Ir* ^P1195L/wt^ males or females with 8- to 20-week-old C57BL6/NCrSlc males or females, we generated two cohorts composed of 42 *Ir* ^P1195L/wt^ males and 64 *Ir* ^wt/wt^ males, and 50 *Ir* ^P1195L/wt^ females and 60 *Ir* ^wt/wt^ females. Male or female mice were randomly divided into four mice per a regular cage. We checked the mice daily and counted the number of dead mice. The body weights of mice were measured at 4-week intervals from 4-weeks after birth until death. 

### 2.3. Histopathological Analysis

Mice were sacrificed via cervical dislocation. The pancreas was removed and fixed with mild formalin solution, and tissue samples were embedded in paraffin. Four-*μ*m-thick sections were stained with hematoxylin and eosin. 

### 2.4. Glucose Metabolism

 The blood glucose concentrations were determined in fasting mice (3-, 12-, 18-, and 25-months old) using an automatic monitor, the Glucocard (Arkray, Kyoto, Japan). The serum insulin levels were measured in 4-, 9-, and 25-month-old mice. Serum obtained from nonfasting or fasting mice was analyzed for insulin using a Mouse Insulin ELISA kit (Shibayagi, Shibukawa, Japan). During glucose tolerance tests, mice fasted overnight and then received intraperitoneally injections of 2 g/kg body weight of 20% D-glucose. The blood glucose concentrations were determined in whole blood obtained from the tail at 0, 15, 30, 60, and 120 minutes after the glucose injection. For the insulin tolerance tests, mice were injected intraperitoneally with 1 U/kg body weight of insulin (Eli Lilly, Indianapolis), then the blood glucose concentrations were measured at 0, 15, 30, and 60 minutes after the injection. The serum adiponectin concentrations were measured in nonfasting mice (4-month-old) using a Mouse/Rat Adiponectin ELISA kit (Otsuka Pharmaceutical, Tokyo, Japan). 

### 2.5. Adipose Tissues and Bone Examination

Mice were sacrificed at 20 months of age and scanned three times using a Lunar PIXImus2 densitometer (GE Healthcare Lunar, Madison, Wis, USA). All mice were fasted for 3 hours before the DEXA. 

### 2.6. Respirometry Monitoring Using Metabolic Cages

Oxygen consumption (VO_2_) and carbon dioxide excretion (VCO_2_) values were measured in 5-month-old male mice (*n* = 3) using an O_2_/CO_2_ metabolism measuring system for small animals (MK-5000RQ, Muromachi Kikai). The mice were isolated in a semisealed cage, and the inner air was aspirated at a constant volume/min (approx., 0.65–0.70 l/min). The concentrations of O_2_ and CO_2_ in the aspirated air were measured per minute at intervals of 3 minutes, and automatically corrected using standard O_2_ and CO_2_ values. The respiratory quotient (RQ) values were calculated by the VCO_2_/VO_2_. 

## 3. Results

### 3.1. *Ir* ^P1195L/wt^ Mice Have a Normal Lifespan

**Figure 1:**
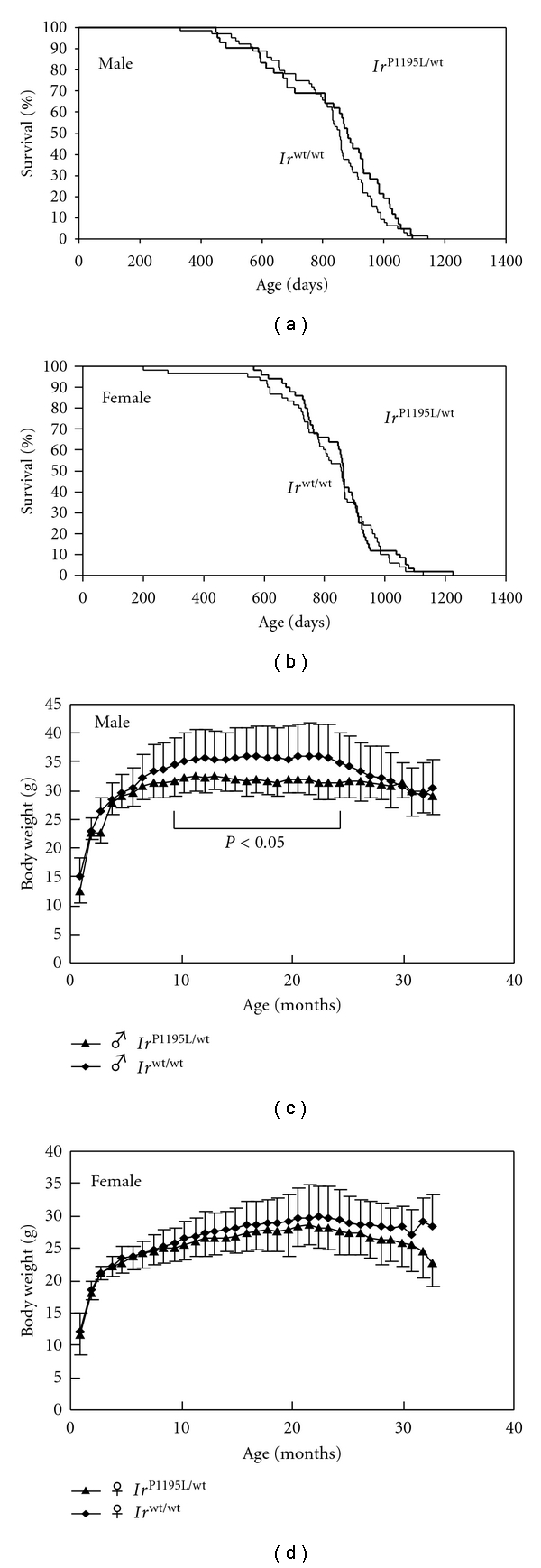
The survival curves and body weights of *Ir* ^P1195L/wt^ mice. (a) The Kaplan-Meier survival curves of *Ir* ^P1195L/wt^ male (*n* = 42) and *Ir* ^wt/wt^ male (*n* = 64) mice (generalized Wilcoxon test, *P* = 0.33). Bold and thin lines indicate *Ir* ^P1195L/wt^ and *Ir* ^wt/wt^ mice, respectively. (b) The Kaplan-Meier survival curves of *Ir* ^P1195L/wt^ female (*n* = 50) and *Ir* ^wt/wt^ female (*n* = 60) mice (generalized Wilcoxon test, *P* = 0.42). (c, d) The body weights of male (c) and female (d) mice. The *Ir* ^P1195L/wt^ male mice (*n* = 28) showed significantly decreased body weight compared to that of *Ir* ^wt/wt^ male mice (*n* = 36) from 9 to 25 months of age, while the *Ir* ^P1195L/wt^ female mice (*n* = 39) failed to show any significant difference compared to the *Ir* ^wt/wt^ female mice (*n* = 47). Closed triangles and closed diamonds indicate *Ir* ^P1195L/wt^ and *Ir* ^wt/wt^ mice, respectively.

In order to evaluate the lifespan of *Ir* ^P1195L/wt^ mice, we compared the 50% survival and maximum lifespan of *Ir* ^P1195L/wt^ mice with those of wild-type (*Ir* ^wt/wt^) mice. As shown in Figure 1(a), the *Ir* ^P1195L/wt^ male mice failed to show an extended lifespan compared to the *Ir* ^wt/wt^ male mice. Likewise, the *Ir* ^P1195L/wt^ female mice also failed to show any increase in survival compared to the *Ir* ^wt/wt^ female mice (Figure 1(b)). A Kaplan-Meier analysis showed that the 50% survival of *Ir* ^P1195L/wt^ male and *Ir* ^wt/wt^ male mice was 883 days (29.0 months) and 855 days (28.1 months), respectively (Figure 1(a)). In females, the 50% survival of *Ir* ^P1195L/wt^ and *Ir* ^wt/wt^ mice was 863 days (28.4 months) and 856 days (28.1 months), respectively (Figure 1(b)). The maximum lifespans of the *Ir* ^wt/wt^ male and female mice were 1,144 days (37.6 months) and 1,226 days (40.3 months), respectively. Those of the *Ir* ^P1195L/wt^ male and female mice were 1,092 days (35.9 months) and 1,126 days (37.0 months), respectively (Figures 1(a) and 1(b)). The results demonstrated that the introduction of an IR mutation does not extend the lifespan of male and female mice (Figures 1(a) and 1(b)).

Since the *Ir* ^P1195L/wt^ male and female mice exhibited a reduction in body weight at 4 months of age compared to the wild type mice [19], we measured the body weight from 1 to 32 months. The body weight of *Ir* ^P1195L/wt^ male mice was lower than that of the *Ir* ^wt/wt^ mice from 9 months to 25 months, while the *Ir* ^P1195L/wt^ female mice failed to exhibit any difference in body weight compared to the wild-type mice throughout their entire lifespan (Figures 1(c) and 1(d)). 

### 3.2. *Ir* ^P1195L/wt^ Mice Exhibit Hyperinsulinemia but Do Not Develop Hyperglycemia

**Figure 2:**
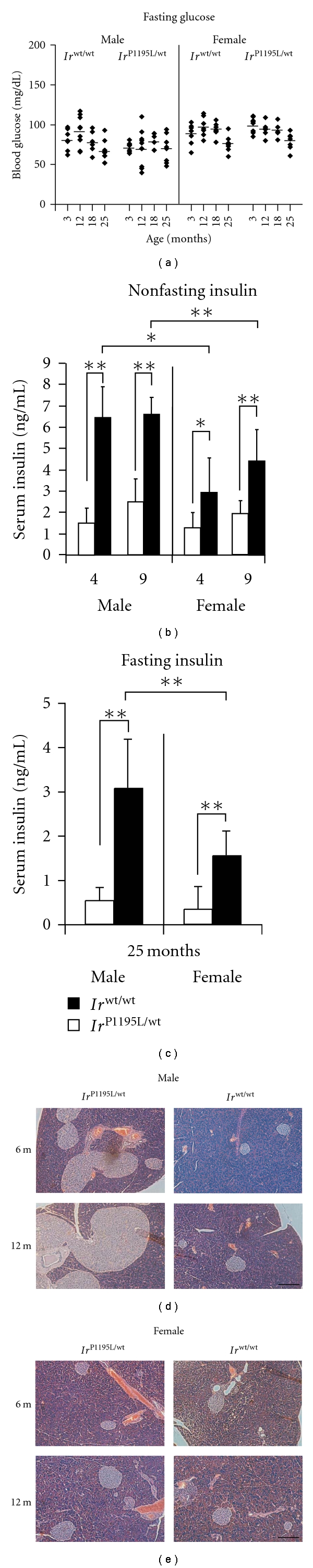
The blood glucose and serum insulin concentrations in *Ir* ^P1195L/wt^ mice. (a) The blood glucose concentrations of fasting mutant male and female mice were assessed. All *Ir* ^P1195L/wt^ mice exhibited blood glucose concentrations within the same range as the *Ir* ^wt/wt^ mice (*n* = 6–10 for each genotype). (b) The serum insulin concentrations were determined in the nonfasting state of 4- and 9-month-old *Ir* ^wt/wt^ and *Ir* ^P1195L/wt^ mice (*n* = 7 for each genotype). (c) The serum insulin concentrations were determined in the fasting state of 25-month-old *Ir* ^wt/wt^ and *Ir* ^P1195L/wt^ mice (*n* = 5 for each genotype). The insulin concentrations were significantly increased in both female and male *Ir* ^P1195L/wt^ mice compared with the *Ir* ^wt/wt^ mice. (d, e) The histochemical analyses of the pancreas of *Ir* ^P1195L/wt^ mice. (d) The *Ir* ^P1195L/wt^ male mice exhibited extensive enlargement of the Langerhans islets compared to *Ir* ^wt/wt^ mice. (e) In addition, the size of islets in *Ir* ^P1195L/wt^ female mice was larger than in *Ir* ^wt/wt^ female mice. The scale bars indicate 200 *μ*m. *P < 0.05, **P < 0.005 by Student's *t-*test.

In order to determine whether the suppression of IR signaling impairs glucose metabolism in aged male and female mice, we measured the blood glucose (Figure 2(a)) and serum insulin (Figures 2(b) and 2(c)) levels in the *Ir* ^P1195L/wt^ male and female mice. In a fasting condition, the *Ir* ^P1195L/wt^ male and female mice did not develop hyperglycemia at any point throughout their entire lifespan (3 to 25 months of age, Figure 2(a)). However, the serum insulin concentrations in the non-fasting state were significantly increased in 4- and 9-month-old male and female mutant mice compared to *Ir* ^wt/wt^ mice (Figure 2(b)). Furthermore, we also found increased insulin concentrations in aged *Ir* ^P1195L/wt^ males and females compared to the *Ir* ^wt/wt^ male and female mice under a fasting condition (Figure 2(c)). Although we failed to detect any gender differences in the insulin concentrations of *Ir* ^wt/wt^ mice, the insulin concentrations of aged *Ir* ^P1195L/wt^ males were significantly increased compared to aged *Ir* ^P1195L/wt^ females (Figures 2(b) and 2(c)). The histological analysis showed the enlargement of Langerhans islets in the pancreas of *Ir* ^P1195L/wt^ mice at 6 and 12 months of age (Figures 2(d) and 2(e)). In particular, *Ir* ^P1195L/wt^ male mice exhibited an extensive hyperplasia of the islets. These results suggest that aged *Ir* ^P1195L/wt^ mice maintained hyperinsulinemia, due to the proliferation of *β*-cells in islets, and did not develop hyperglycemia during aging. 

**Figure 3:**
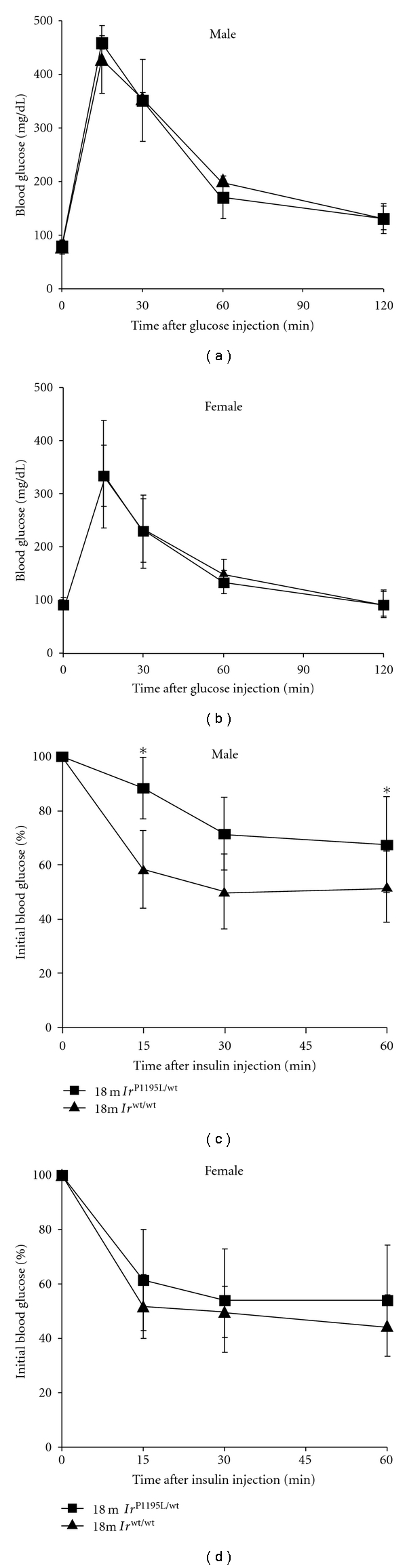
The results of the glucose and insulin tolerance tests in aged *Ir* ^P1195L/wt^ mice. (a, b) Glucose tolerance tests were performed on 18-month-old *Ir* ^wt/wt^ and *Ir* ^P1195L/wt^ mice (*n* = 5 for each genotype). (c, d) Insulin tolerance tests were performed on 18-month-old *Ir* ^wt/wt^ and *Ir* ^P1195L/wt^ mice (*n* = 5 in each genotype). *P < 0.05 by Student's *t-*test.

In order to determine the physiological compensation for the mutation of the IR gene, we assessed the glucose and insulin tolerances of *Ir* ^P1195L/wt^ mice at 18 months of age. We previously reported that young *Ir* ^P1195L/wt^ male mice showed glucose intolerance and reduced insulin sensitivity, while *Ir* ^P1195L/wt^ females did not show these tolerances, in spite of hyperinsulinemia [19]. In the glucose tolerance test, the 18-month-old *Ir* ^P1195L/wt^ male and female mice both showed a normal glucose tolerance (Figures 3(a) and 3(b)). On the other hand, aged *Ir* ^P1195L/wt^ male mice had impaired insulin sensitivity (Figure 3(c)), while aged *Ir* ^P1195L/wt^ females and *Ir* ^wt/wt^ females reacted similarly to insulin administration (Figure 3(d)). Based on the data about glucose metabolism in young and aged mice, we suggested that *Ir* ^P1195L/wt^ male mice maintained reduced insulin sensitivity during aging, but that the *Ir* ^P1195L/wt^ female mice did not. 

### 3.3. *Ir* ^P1195L/wt^ Mice Show a Reduction of Adipose Tissue and Enhancement of Bone Density

**Figure 4:**
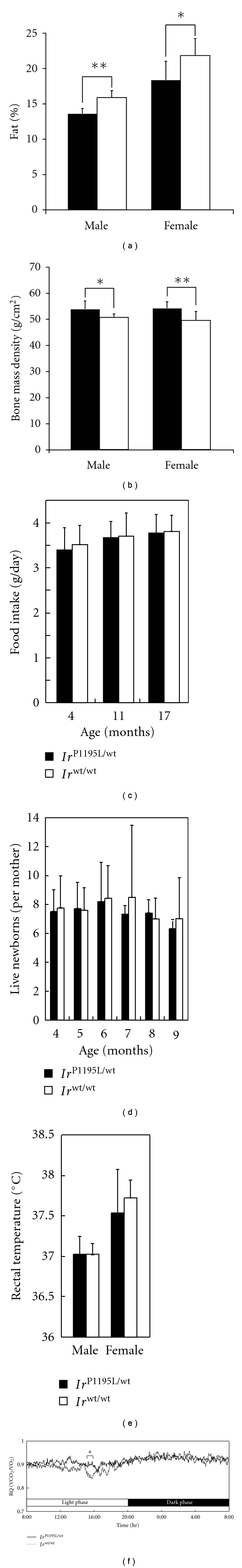
Adiposity, bone density, food intake, reproduction, rectal temperature, and respiratory quotients of the *Ir* ^P1195L/wt^ mice. (a) Both male and female *Ir* ^P1195L/wt^ mice showed a significantly decreased fat ratio compared to *Ir* ^wt/wt^ mice at 20 months of age. (b) Both male and female *Ir* ^P1195L/wt^ mice showed significantly increased bone density compared to the *Ir* ^wt/wt^ mice at 20 months of age. Aged *Ir* ^P1195L/wt^ male (*n* = 6), *Ir* ^P1195L/wt^ female (*n* = 6), *Ir* ^wt/wt^ male (*n* = 6), and *Ir* ^wt/wt^ female (*n* = 5) mice were evaluated by DEXA. (c) The food intake of *Ir* ^P1195L/wt^ male and *Ir* ^wt/wt^ male mice at 4, 11, and 17 months of age. No significant difference was observed between the food consumption by *Ir* ^P1195L/wt^ (*n* = 10) and *Ir* ^wt/wt^ (*n* = 12) mice. (d) The reproduction rate of *Ir* ^P1195L/wt^ female mice. No significant difference was observed between *Ir* ^P1195L/wt^ and *Ir* ^wt/wt^ mice (at least 3 mice per genotype). (e) The rectal temperatures of *Ir* ^P1195L/wt^ and *Ir* ^wt/wt^ mice at 4 months of age. No significant differences were observed in the temperatures between the *Ir* ^P1195L/wt^ and *Ir* ^wt/wt^ mice (*n* = 4 for each genotype). *P < 0.05, **P < 0.005 by Student's *t-*test. (f) The mean time course of respiratory quotients (RQ = VCO_2_/VO_2_) of male mice at 5 months of age. The values represent the mean of 24 hours of measurements from each animal (*n* = 3, male). *P < 0.05 by Student's *t-*test.

In the previous study, we showed a reduction of perigonadal adipose tissues in *Ir* ^P1195L/wt^ male and female mice [19]. To assess the overall distribution of adipose tissues in mutant mice at 20 months of age, we measured the adipose mass by dual-energy X-ray absorptiometry (DEXA). The *Ir* ^P1195L/wt^ male and female mice showed a significantly reduced adipose mass (Figure 4(a)). Since the circulating adiponectin levels negatively correlate with visceral adiposity in humans [21], we measured the serum adiponectin concentrations in non-fasting *Ir* ^P1195L/wt^ mice. The adiponectin concentrations in the *Ir* ^P1195L/wt^ mice were not increased compared to *Ir* ^wt/wt^ mice (male; 23.7 ± 2.8 versus 27.0 ± 7.6 *μ*g/mL, female; 44.8 ± 5.0 versus 42.3 ± 3.3 *μ*g/ml, resp.,) at 4 months of age, suggesting that the reduced adiposity induced by altered IR signaling might be insufficient to enhance the secretion of adiponectin from adipose tissues in *Ir* ^P1195L/wt^ mice.

Since osteoblasts and adipocytes are differentiated from common progenitor cells in the bone marrow [22–24], we assumed that the common progenitor cells of *Ir* ^P1195L/wt^ mice had differentiated into osteoblasts, which would lead to enhanced bone formation instead of generating adipose tissue in these mice. The DEXA analysis demonstrated that there was a significant increase in the bone density in aged *Ir* ^P1195L/wt^ mice (Figure 4(b)). Interestingly, 4-month-old *Ir* ^P1195L/wt^ mice failed to exhibit any significant difference in bone density compared to 4-month-old *Ir* ^wt/wt^ mice (data not shown). These results suggested that one of the beneficial effects of the *Ir* ^P1195L/wt^ genotype in the later stage of life is due to the reduction of adipose mass associated with the enhancement of bone density. 

### 3.4. *Ir* ^P1195L/wt^ Mice Show Normal Food Intake, Reproduction Rates, Rectal Temperatures, and Metabolism

Since dietary restriction can prolong the lifespan of animals [25], we compared the food intake of *Ir* ^P1195L/wt^ mice with that of *Ir* ^wt/wt^ mice. We failed to detect any significant difference in food intake between the *Ir* ^P1195L/wt^ and *Ir* ^wt/wt^ mice (Figure 4(c)), suggesting that dietary restriction did not affect the lifespan of the *Ir* ^P1195L/wt^ mice. Since long-lived dwarf mice display reduced fecundity [9], we measured the reproduction rate in *Ir* ^P1195L/wt^ female mice. As shown in Figure 4(d), the results indicated that the *Ir* ^P1195L/wt^ mice reproduced to generate a number of pups comparable to the number produced by *Ir* ^wt/wt^ female mice. 

 A relationship between low body temperature and lifespan has been reported in long-lived Ames dwarf mice and rhesus monkeys on dietary restriction [26–28]. Therefore, we measured the rectal temperature of *Ir* ^P1195L/wt^ mice. We failed to detect any significant differences in the rectal temperatures between *Ir* ^P1195L/wt^ and *Ir* ^wt/wt^ mice (Figure 4(e)). Since metabolism may have an important role in aging, we also analyzed voluntarily running distance to evaluate the physical activity in *Ir* ^P1195L/wt^ mice. However, we did not observe any substantial differences in running distance for 14 days between *Ir* ^P1195L/wt^ male and *Ir* ^wt/wt^ male mice (data not shown). We also measured the VO_2_ and VCO_2_ of the mice in metabolic cages. Although the respiratory quotient value of the *Ir* ^P1195L/wt^ males was slightly increased in the light phase, the *Ir* ^P1195L/wt^ mice showed comparable metabolism to *Ir* ^wt/wt^ male mice (Figure 4(f)). These results suggested that the *Ir* ^P1195L/wt^ mice did not have modulation in their metabolism, such as reduced food intake, lower body temperature, or any differences in their physical activity, respiratory quotient, or reproductive activities. 

## 4. Discussion

### 4.1. The Biological Roles of Insulin Signaling in Adipose Tissue for Lifespan Extension

Bluher et al. reported that specific deletion of the IR gene in adipose tissues extended the lifespan of FIRKO mice [18]. FIRKO mice also showed reduced adiposity, as well as normal food intake. The authors suggested in that paper that leanness, not dietary restriction, is a key contributor to an extended lifespan [18]. Since IR signaling was completely downregulated in the adipose tissues of FIRKO mice, but was normal in other tissues, the loss of insulin signaling might confer a reduction of fat mass in adipose tissues. In the present study, we observed that the *Ir* ^P1195L/wt^ mice displayed a reduction in fat mass with normal food intake (Figures 4(a) and 4(d)), indicating that the *Ir* ^P1195L/wt^ mice showed similar phenotypes to the FIRKO mice with respect to their adiposity. However, the *Ir* ^P1195L/wt^ mice failed to show an extended lifespan, in contrast to the FIRKO mice (Figures 1(a) and 1(b)). Although the differences in the beneficial effects on lifespan in these mutant mice is still unclear, we believe that there are three possible reasons for these differences, as described below. 

First, the reduced fat mass in *Ir* ^P1195L/wt^ mice was not sufficient to extend the lifespan of these mice, while the reduced level in the FIRKO mice was sufficient to produce a lifespan change. In fact, we failed to observe any difference in the serum adiponectin concentration between the *Ir* ^P1195L/wt^ and wild-type mice. Second, in contrast to the IR mutation of *Ir* ^P1195L/wt^ mice, the IR insufficiency, specifically in the adipose tissues of the FIRKO mice, might modulate their lifespan by an independent mechanism. The insulin signaling in adipose tissues of FIRKO mice might promote the differential regulation of the biosynthesis of adipokines such as adiponectin, leptin, tumor-necrosis factor-*α*, resistin, and retinol binding protein-4. Third, FIRKO mice exhibited tissue-specific reduced insulin signaling in adipose tissues. In contrast, global insulin resistance due to a systemic IR mutation might inhibit the beneficial effects of a reduced fat mass on lifespan extension in mice. 

### 4.2. IGF-1 Receptor Signaling May Dominantly Regulate the Lifespan in Mice

It is unclear whether the IR or IGF-1R corresponds to the function of *daf-2* in *C. elegans* with regard to the lifespan regulation in vertebrates. Holzenberger et al. reported that both male and female IGF-1R gene heterozygous mice showed an extended lifespan, with normal fertility, and that they also showed an increased resistance to oxidative stress [17]. Since the mutation introduced in the IR gene presented in this paper showed a dominant negative effect, and the tyrosine kinase activities were severely down-regulated, it is difficult to directly compare its phenotype with IGF-1R-deficient mice that show haploinsufficiency in IGF-1R signaling. Although decreased IGF-1R signaling prolonged the lifespan in IGF-1R mutant mice, altered IR signaling failed to extend the lifespan in *Ir* ^P1195L/wt^ mice. Another line of evidence for the importance of IGF-1R signaling in lifespan regulation is that long-lived dwarf mice display low levels of GH and circulating IGF-1 [29, 30]. Furthermore, targeted inactivation of the GH receptor also showed a decrease in the circulating IGF-1 level, and increased lifespan [11]. These results revealed that IGF-1 signaling might dominantly regulate the lifespan in mice, while insulin signaling might mainly regulate glucose and energy metabolism, rather than lifespan. 

### 4.3. Comparison of * Ir * Mutant Mice with Other Long-Lived Mutant Mice, Including Klotho Tg, * Irs1*, * Irs2*, and S6K Mutant Mice, on Lifespan Extension

Kurosu et al. reported that overexpression of the *Klotho* gene extends the lifespan in male and female mice [31]. Two lines of *Klotho* Tg mice also showed reduced fecundity in females and insulin resistance in both genders. When the soluble Klotho protein was intraperitoneally administered to wild-type mice, the insulin and IGF-1 sensitivity of the mice was impaired, indicating that increased Klotho protein in the blood induced insulin resistance, as well as IGF-1 resistance. Since the *Ir* ^P1195L/wt^ mice also showed insulin resistance, but not IGF-1 resistance, IGF-1 resistance might be necessary for the lifespan extension in mice. This result is consistent with the reduced IGF-1 signaling observed in *Igf1r* ^+/−^ and long-lived dwarf mice. 

Recently, Taguchi et al. and Selman et al. reported that mice with mutations in *Irs1* and *Irs2*, components of the insulin/IGF-1 signaling pathway, showed an extended lifespan and reduced insulin sensitivity [32, 33]. Selman et al. also reported that deletion of ribosomal S6 protein kinase (S6K), a component of the nutrient-responsive mTOR (mammalian target of rapamycin) signaling pathway, led to an increased lifespan and loss of insulin sensitivity [34]. Taken together with these reports, our results suggest that reduced insulin sensitivity has a limited effect on the lifespan in mice. 

### 4.4. Oxidative Stress and Lifespan in *Ir* ^P1195L/wt^ Mice

Long-lived nematode and fly mutants with altered insulin signaling generally acquire enhanced resistance to oxidative stress [3, 7, 8, 35]. Furthermore, long-lived dwarf, *Igf1r* ^+/−^, and p66^shc-/−^ mice also showed enhanced resistance to paraquat [9, 17, 36]. In our previous study, we reported that *Ir* ^P1195L/wt^ male and female mice also showed significantly enhanced resistance to oxidative stress, such as hyperoxia and paraquat [19]. Furthermore, *Ir* ^P1195L/wt^ female mice survived longer than *Ir* ^P1195L/wt^ male mice in an 80% oxygen chamber, suggesting that sex hormones modulate the resistance to oxidative stress in mice. We also demonstrated that estrogen modulated the resistance to oxidative stress by estrogen administration in male and ovariectomy in female mice. In the present study, however, the *Ir* ^P1195L/wt^ female mice failed to show any increase in survival under normoxia (Figure 1(c)). Likewise, the *Ir* ^P1195L/wt^ male mice showed a normal lifespan (Figures 1(a) and 1(b)). These results indicate that the resistance to oxidative stress is not correlated with the lifespan in mice. Although we are unable to exclude the possibility that other limiting factors modulated the lifespan in *Ir* ^P1195L/wt^ mice, we suggest that the enhanced resistance to oxidative stress is not a major determinant for lifespan extension in mice. 

### 4.5. *Ir* ^P1195L/wt^Mice Show a Normal Lifespan, without Developing Hyperglycemia

Mutations in the IR gene cause insulin resistance syndromes, such as leprehaunism, Rabson-Mendenhall syndrome, and type A insulin resistance [37, 38]. We introduced a mutation substituting a Leu^1195^ for the Pro^1195^ residue in the *β* chain of *Ir* ^P1195L/wt^ mice. This mutation corresponds to a homologous mutation substituting a Leu^1178^ for the Pro^1178^ residue of the human IR in patients with type A insulin resistance found in Japanese and British patients [39, 40]. A Japanese patient with the orthologous mutation showed obesity, moderate insulin resistance, acanthosis nigricans, and hyperandrogenism with normal glucose tolerance [39]. The authors of that paper suggested that insulin resistance and the other clinical features observed in the patient were due to obesity rather than the mutation in the IR gene [39]. Interestingly, Krook et al. reported that a type A Cam-3 patient with an orthologous mutation presented with oligomenorrhea, hirsutism, and acanthosis nigricans at 13 years of age [40]. She subsequently developed hyperglycemia. Moreover, the authors described in this paper that the patient had inherited the IR mutation from her father, who was clinically normal but had a moderate elevation of his fasting insulin. The patient's mother and sister also had moderate hyperinsulinemia, which suggests that there was a second defect in this patient [40]. In this context, we speculated that there was a heterozygous mutation in the father or mother of the British patient who had hyperinsulinemia without developing hyperglycemia, while the second defect in the patient led to severe insulin resistance and hyperglycemia. Based on these reports and our present data, we suggested that the heterozygosity of the orthologous mutation with the kinase domain of the IR gene leads to moderate insulin resistance with hyperinsulinemia, but does not lead to the development of hyperglycaemia in mice or humans.

Kido et al. reported that genetic modifiers of insulin resistance influence the phenotype in IR-deficient mice [41]. On the genetic background of B6 mice, the haploinsufficiency of the IR gene caused mild hyperinsulinemia. In contrast, on the genetic background of 129/Sv mice, the same mutation caused severe hyperinsulinemia, suggesting that the 129/Sv strain harbors alleles that interact with the IR mutation and predispose these mice to insulin resistance [41]. Interestingly, these data were obtained for males, while females did not develop hyperinsulinemia on either background. These studies also indicated that 5-10% of IR^+/−^ mice on the B6 background developed hyperglycemia, while 25% of IR^+/−^ mice on the 129/Sv background developed hyperglycemia [41, 42]. Thus, the B6 strain is relatively resistant to the deleterious effects of the haploinsufficiency of the IR gene. Since we developed our *Ir* ^P1195L/wt^ mice on a B6 genetic background, further analyses in mice of a different background may provide us with information about the genetic modifier(s) and help evaluate the pathological effects of the IR mutation on the development of hyperglycemia. 

## 5. Conclusion

In the present study, we showed that *Ir* ^P1195L/wt^ mice showed a normal lifespan, as well as hyperinsulinemia, and they did not develop hyperglycemia throughout their lifespan. This study provides evidence that insulin signaling does not make a major contribution to regulating the lifespan in mammals. A further analysis of altered insulin signaling using *Ir* ^P1195L/wt^ mice, especially those developed on another background, should provide us with a useful strategy for the prevention of age-associated diseases, such as type 2 DM in humans.
